# A Single Dose of 5-MeO-DMT Stimulates Cell Proliferation, Neuronal Survivability, Morphological and Functional Changes in Adult Mice Ventral Dentate Gyrus

**DOI:** 10.3389/fnmol.2018.00312

**Published:** 2018-09-04

**Authors:** Rafael Vitor Lima da Cruz, Thiago C. Moulin, Lyvia Lintzmaier Petiz, Richardson N. Leão

**Affiliations:** ^1^Neurodynamics Lab, Brain Institute, Federal University of the Rio Grande do Norte, Natal, Brazil; ^2^Institute of Medical Biochemistry, Federal University of Rio de Janeiro, Rio de Janeiro, Brazil; ^3^Developmental Genetics, Department of Neuroscience, Uppsala University, Uppsala, Sweden

**Keywords:** 5-MeO-DMT, adult neurogenesis, patch clamp, psychedelics, dentate gyrus granule cells

## Abstract

The subgranular zone (SGZ) of dentate gyrus (DG) is one of the few regions in which neurogenesis is maintained throughout adulthood. It is believed that newborn neurons in this region encode temporal information about partially overlapping contextual memories. The 5-Methoxy-N,N-dimethyltryptamine (5-MeO-DMT) is a naturally occurring compound capable of inducing a powerful psychedelic state. Recently, it has been suggested that DMT analogs may be used in the treatment of mood disorders. Due to the strong link between altered neurogenesis and mood disorders, we tested whether 5-MeO-DMT is capable of increasing DG cell proliferation. We show that a single intracerebroventricular (ICV) injection of 5-MeO-DMT increases the number of Bromodeoxyuridine (BrdU+) cells in adult mice DG. Moreover, using a transgenic animal expressing tamoxifen-dependent Cre recombinase under doublecortin promoter, we found that 5 Meo-DMT treated mice had a higher number of newborn DG Granule cells (GC). We also showed that these DG GC have more complex dendritic morphology after 5-MeO-DMT. Lastly, newborn GC treated with 5-MeO-DMT, display shorter afterhyperpolarization (AHP) potentials and higher action potential (AP) threshold compared. Our findings show that 5-MeO-DMT affects neurogenesis and this effect may contribute to the known antidepressant properties of DMT-derived compounds.

## Introduction

Psychoactive tryptamines are a class ofmolecules that act as a neurotransmitter in the vertebrate brain (Jacob and Presti, 2005). N,N-dimethyltryptamine, (DMT) and analogues, are closely related to 5-methoxy- N,N-dimethyltryptamine (5-MeO-DMT), they can be found in a great variety of plants in South America, with an even greater diversity of chemical analogs (Geyer et al., 2010; Greene, 2013). 5-MeO-DMT is a serotonin agonist that acts in a non-selective manner in 5-HT2A >5-HT2C >5-HT1A receptors (Szabo et al., 2014). However, the N-N-DMT has been reported elsewhere to also acts in many glutamate, dopamine, and acethylcholine receptors (Carbonaro and Gatch, 2016). It would be interesting to know whether the 5-MeO-DMT have the same effect as its analogue on those receptors. The 5-MeO-DMT is analogous of the N,N-DMT, one of the main active ingredients of *Ayahuasca*, a millenarian decoction used as a sacrament by south American indigenous tribes, known to induce powerful hallucinogenic states when administered with monoamine oxidase inhibitors (MAOI; Araújo et al., 2015). At present, *Ayahuasca* is used by many syncretic churches ritualistically, as a way to heal many physical and mental illnesses with or without scientific knowledge about the effects (Frecska et al., 2016). Recent studies also suggest that *Ayahuasca* can potentially treat recurrent depression (Osório Fde et al., 2015; Sanches et al., 2016) even in a placebo controlled frame (Palhano-Fontes et al., 2018).

Deficits in adult neurogenesis are associated with the physiopathology of depression and modulation of neurogenesis is behind the action of several antidepressants (Santarelli et al., 2003). Serotonin reuptake inhibitors, for example, rescue normal neurogenesis levels in animal models of depression (Duman et al., 2001; Lledo et al., 2006; Perera et al., 2007; Sahay and Hen, 2007; Hill et al., 2015; Noto et al., 2016). Adult neurogenesis is known to occur in two sites in the brain, the subgranular zone (SGZ) of the dentate gyrus (DG) and the subventricular zone (SVZ) of the lateral ventricle (Gould, 2007). There is some debate if SVZ neurogenesis responds or not to mood disorders and psychoactive drugs (Encinas et al., 2006; Hanson et al., 2011; Ohira and Miyakawa, 2011) but the effect of mood disorders in SGZ Radial glial Like cell (RGL) proliferation and neuronal survivor is prolifically described (Castrén and Hen, 2013). Interestingly, alkaloids from one of the plants used in the *Ayahuasca* brew stimulate neurogenesis *in vitro* (Morales-García et al., 2017); however, it is not known whether *in vivo* adult neurogenesis is affected by psychoactive tryptamines.

In this study we tested if a single dose of 5-MeO-DMT affects neurogenesis in mice. We found that after a single intracerebroventricular (ICV) injection of 5-MeO-DMT, cell proliferation in the DG was significantly larger in comparison to saline. Moreover, the number of DCX::tdTom+ cells are also higher for experimental group, these same DG granule cells (GC) show more complex dendritic trees when compared to control animals. Finally, we found that afterhyperpolarization (AHP) potential duration where shorter and action potential (AP) threshold higher in newborn neurons from mice treated with 5-MeO-DMT.

## Materials and Methods

### Ethics Statement

This study was carried out in accordance with the recommendations of the National Council for the Control of Animal Experimentation (CONCEA) in Brazil. The protocol was approved by the local animal care institution of the Federal University of Rio Grande do Norte (Protocols 041/2014 and 015.004/2017).

### Animals

Adult C57BL6J and DCX-CreER^T2^::tdTom^lox/lox^ transgenic (Zhang et al., 2010; Leão et al., 2012) mice from both sex aged between 55–70 days were used in this study. Animals were housed under a 12 h light/12 h dark cycle. Food and water *ad libitum*.

### 5-MeO-DMT Treatment

Animals anesthetized with isoflurane (3%–5% L/min for induction and 1%–3% L/min for maintenance; Gargiulo et al., 2012) received a single ICV injection of 1 μL 5-MeO-DMT solution (100 μg 5-MeO-DMT in 10% DMSO/90% saline) prepared fresh (Commissaris and Davis, 1982; Galvao et al., 2014) control groups received 1 μL of 10% DMSO in saline (stereotaxic coordinates: 0.3 mm AP, 1.0 mm ML and 2.8 mm DV; DeVos and Miller, 2013).

### BrdU Labeling, Tamoxifen Treatment, Cryopreservation and Slicing

Ten to fifteen minutes after 5-MeO-DMT or saline ICV injections, animals (under anesthesia) received 50 mg/kg of Bromodeoxyuridine (BrdU, Sigma) intraperitoneally (IP) diluted in saline. For proliferation assays mice were sacrificed (overdose of ketamine 130 mg/kg mixed with 8 mg/kg xylazin) and perfused with PBS followed by paraformaldehyde (PFA) 12 h after BrdU injection. To induce recombination in DCX-CreER^T2^::tdTom^lox/lox^ animals were treated 100 μg/g/day of tamoxifen IP 3 days after ICV injections. These animals were either perfused for histology following the same slicing and freezing protocol for BrdU staining, or anesthetized and had the brains removed for patch clamp experiments (see below). For histology experiments, brains from PFA perfused animals were removed and postfixed in 4% PFA overnight. Brains were then washed in PB 0.1 M (pH = 7.4) for 10 min then immersed in graded sucrose solutions (10/20/30%) for cryopreservation, then snap frozen by immersion into −80°C isopropyl alcohol and stored in −80°C freezer for posterior cryosectioning. Forty micrometer horizontal hippocampal sections were cut in a cryostat (Thermo Microm HM 550) for BrdU immunohistochemistry and DCX::tdTom count. Eight slices from each animal, containing both ventral hippocampi, were gathered, spaced with 200 μm between them (every fifth slice were collected) to avoid sample the same population twice. In DCX-CreER^T2^::tdTom^lox/lox^ cell counting were performed in a single hemisphere as the other hemisphere was used for patch clamp experiments (in order to reduce the number of animals used for the experimental purpose, in accordance with local guidelines of the the Brazilian guidelines for laboratory animal welfare).

### BrdU Immunohistochemistry

Hippocampal slices were washed with PBS (pH = 7.4) for 10 min at room temperature (RT), then placed for 30 min into HCl 2N at 37°C to open DNA double strand, washed again in PBS, transferred to borate buffer (pH = 8.0) at RT for 20 min, then washed in PBS and incubated overnight in primary antibody solution: 10% normal goat serum (NGS; Sigma), 1:500 Rat igG anti-BrdU (Abcam) and 0.3% X-100 triton in PBS solution (Sigma). Slices were then washed in PBS for 10 min and incubated for 2 h in secondary antibody solution: 10% NGS, 1:1,000 rabbit igG anti-rat rabbit F(ab’)2 Anti-Rat IgG H&L conjugated with Alexa Fluor^®^ 488 (Abcam) and 0.3% triton x-100 in PBS. Slices were subsequently washed with PBS solution and incubated in 1:2,000 Hoechst 33425 (ThermoFisher) in PBS 10 mM (nuclei staining), washed in PBS and mounted on N-propyl gallate solution mounting medium. Hippocampal slices were imaged using an epifluorescence upright microscope (ZEISS) with Stereoinvestigator software (MBF Bioscience), BrdU+ cells were manually counted in both hippocampi by an experimenter blinded for groups.

### Clustering Analysis of BrdU+ Cells

After microscopy, images were processed by a personal MATLAB code, where the total number of cells and distances between them were calculated. We computed this data to generate a graph where each cell was considered a node. If the distance between two cells was less than 25 μm, an edge was created between them and its weight was increased as closer they were. We analyzed the final graph using the Girvan-Newman’s modularity algorithm (Girvan and Newman, 2002), Considering clusters as groups of 1–5 cells. We then measured how many clusters were formed and the number of cells within clusters.

### Electrophysiology and Dendritic Morphology Analysis

DCX-CreER^T2^::tdTom^lox/lox^ animals were anesthetized with ketamine hydrochloride (100 mg/kg) and xylazine hydrochloride (8 mg/kg) then intracardially perfused with RT standard cerebrospinal fluid (aCSF; in mM: NaCl 124; KCl, 2.5; NaH_2_PO_4_, 1.2; NaHCO_3_, 24; glucose, 12, 5; CaCl_2_, 2; MgCl_2_, 2). Animals were then decapitated and had their brains removed and then transferred to a vibratome chamber containing ice-cold aCSF, slices with 300 μm thickness were collected in the Vibratome (VT1200, Leica) and transferred to a custom designed 3d printed incubation chamber containing recover NMDG solution (in mM NMDG, 92; KCl, 2.5; NaH_2_PO_4_, 1.25; NaHCO_3_, 30; HEPES, 20; glucose, 25; thiourea, 2; sodium-ascorbate, 5; sodium-pyruvate, 3; CaCl_2_·4H_2_O, 0.5; 10 MgSO_4_·7H_2_, 10; pH controlled to 7.3–7.4 with 2N HCl solution) at 36°C for 15 min, and then again returned to aCSF for at least 1 h at RT prior to recordings, all solutions were continually bubbled with carbogen 95% O_2_ and 5% CO_2_ (White-Martins; Ting et al., 2014). For whole-cell patch clamp recordings the tissue was transfered to a chamber filled with Standard aCSF in a Microscope (ZEISS). Micropipettes were filled with K-gluconate solution (in mM, K-Gluconate, 145; HEPES, 10; EGTA, 1; Mg-ATP, 2; Na2-GTP, 0.3; MgCl_2_, 2; pH 7.3, 290–300 mOsm) GC from DCX-CreER^T2^::tdTom^lox/lox^ mice were identified by fluorescence (543 excitation/580 emission). Current-clamp recordings were obtained using an axopatch amplifier 200B (Molecular Devices) in whole-cell configuration using the winWCP Strathclyde Electrophysiology Software. Two protocols were used in current clamp: 100 ms-long current steps with 50 pA increment ranging from −100 pA to 400 pA and a ramp ranging from −50 pA to 200 pA in 1500 ms. Current clamp data was analyzed using winWCP. Spontaneous excitatory postsynaptic currents (sEPSCs) were recorded in voltage clamp using winECP (free run—cells held at −60 mV). To analyze sEPSCs, events were first detected using a custom Matlab (Mathworks) program (‘pspAnalysis.m’). The program detects EPSCs based on a template using correlation coefficient calculated in a sliding window. The program can be downloaded from https://github.com/cineguerrilha/Neurodynamics. Some hippocampal sections were fixed after slices and used for Sholl analysis. To analyze dendritic morphology, slices from DCX-CreER^T2^::tdTom^lox/lox^ mice, obtained as above, were kept overnight in PFA 0.4% overnight and 40× -amplification pictures were taken using confocal microscopy (Zeiss) to analyze the Tomato-expressing neurons. The images in which was possible to clearly visualize the dendritic arborization were blinded selected to experimental groups. Morphometry was performed using the ImageJ plug-in Simple Neurite Tracer, extracting the number of branches and performing Sholl analysis (Ferreira et al., 2014).

### Statistical Analysis

All data is normal distributed, tested for normality with D’Agostino and Pearson omnibus normality test. Comparisons between groups were made with unpaired *t*-test. For BrdU staining, cell clustering analysis and DCX-CreER^T2^::tdTom^lox/lox^ cell counting, eight sections containing ventral hippocampus was chosen from each animal, dorsal hippocampus and the smallest portion of ventral hippocampus from each animal were excluded from analysis. For mean comparison, the total number of BrdU+ cells or DCX::tdTOM+ cells were accounted from each selected section and summed up, then, the total number per animal were used for unpaired *t*-test. For Sholl analysis of dendritic arborization, two-way ANOVA was performed, with Holm-Sidak’s *post hoc* test comparing each 10 μm-section away from soma for both treatments. All data is presented as mean ± Standard Error Mean (SEM).

## Results

**Figure 1:**
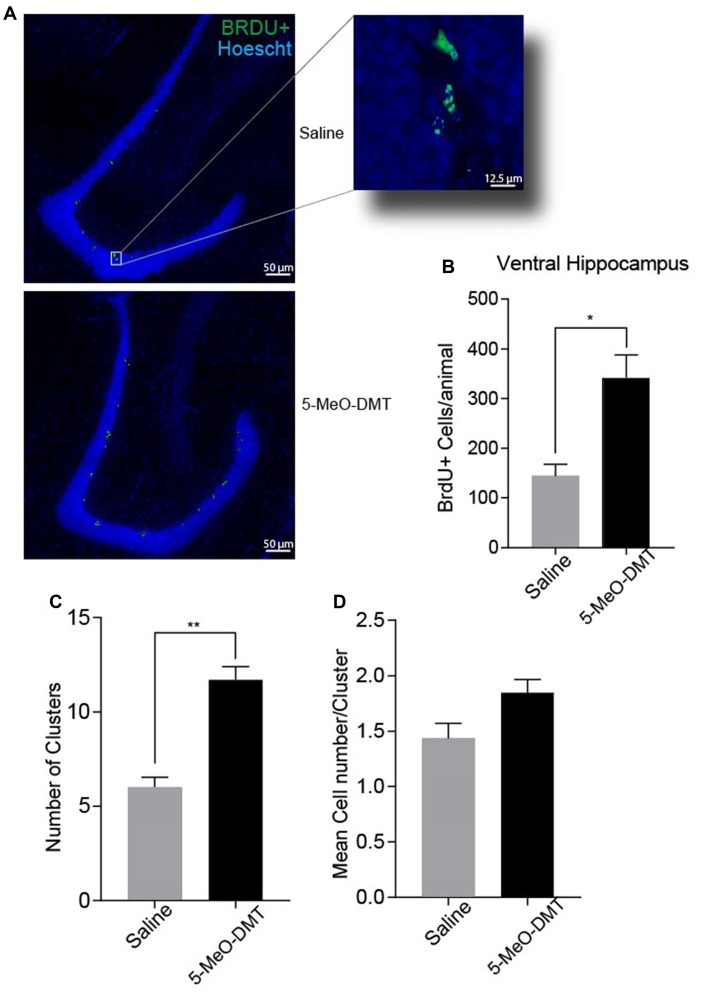
Single dose of5-Methoxy-N,N-dimethyltryptamine (5-MeO-DMT) increases cell proliferation within the dentate gyrus (DG) of adult mice. **(A)** Photomicrography showing representative hippocampal sections Bromodeoxyuridine (BrdU+) cells in green and hoechst 33342 in blue. **(B)** Average number of BrdU+ cells in the adult mice ventral DG. **(C)** Mean number of cells per clusters. **(D)** Mean number of clusters in each group. *p = 0.0029, **p = 0.0002.

In order to check whether a single dose of 100 μg of 5-MeO-DMT increases cell proliferation in the adult DG as other serotonin 5-HT_1A_ agonists can (Encinas et al., 2006), we labeled cells in S phase with BrdU (Taupin, 2007). We found that 5-MeO-DMT treated animals showed a greater number of BrdU+ cells in the ventral DG compared to saline injected controls (saline treated: 155.4 ± 21.71 BrdU^+^ cells per animal—see methods, *n* = 5 mice; 5-MeO-DMT treated: 352.6 ± 41.48 BrdU+ cells per animal, *n* = 5 mice. *p* = 0.0029, unpaired *t*-test, Figures 1A,B). To investigate if the increase in cell proliferation was due to the recruitment of new progenitors or by an enhancement in progenitor division, we analyzed the cluster formation of the BrdU+ cells in DG in control and 5-MeO-DMT injected mice. Clustered cells suggest that they originate from the same progenitor, since newborn cells start to migrate right after proliferation (Brown et al., 2003). The total number of clusters in the ventral DG of 5-MeO-DMT treated animals was greater than saline (saline treated: 5.964 ± 0.5718 clusters per section, eight sections were analyzed per mice, *n* = 5 mice; 5-MeO-DMT treated: 11.7 ± 0.6974 clusters per section, eight sections were analyzed per mice, *n* = 5 mice. *p* = 0.0002, unpaired *t*-test, Figure 1C). Moreover, we found a small difference in the number of cells per cluster between control and 5-MeO-DMT-treated mice trending to significance (saline treated: 1.433 ± 0.1365 cells per cluster, *n* = 5 mice; 5-MeO-DMT treated: 1.846 ± 0.119 cells per cluster, *n* = 5 mice. *p* = 0.0523, unpaired *t*-test, Figure 1D). This data suggests that a greater number of progenitors are being recruited by 5-MeO-DMT.

**Figure 2:**
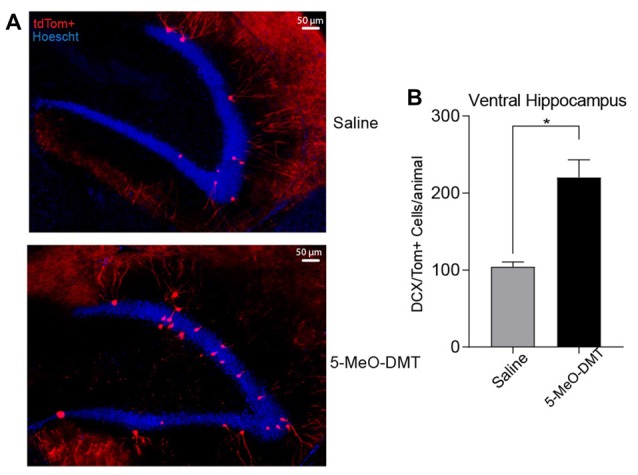
Single dose of 5-MeO-DMT increase the number of new DG granule cells (GC) 21 days after injection. **(A)** Photomicrography showing representative hippocampal sections (DCX::tdTom+ cells in red and hoechst 33342 in blue). **(B)** Average number of DCX::tdTom+ cells per group. *p = 0.0006.

Following the proliferation assay, we tested if the number of newborn DG GC after ICV injection of 5-MeO-DMT is higher, aiming to answer if the 5-MeO-DMT also increase survivability of newborn neurons generated within the hippocampus. Our results indicate that the total number of DCX::tdTom+ cells is higher in the ventral hippocampus of 5-MeO-DMT treated mice (saline treated: 104 ± 6.348 DCX::tdTom+ cells, *n* = 6 mice; 5-MeO-DMT treated 220.3 ± 22.86 DCX::tdTom+ cells, *n* = 6 mice, *p* = 0.0006, unpaired *t*-test, Figures 2A,B). Remembering that for the DCX-CreER^T2^::tdTom^lox/lox^ mice, only a single hemisphere were used, not allowing accurate comparison between proliferation and survivability assays, however, the proportion of difference between the two treatments remain the same for both experimental frames.

**Figure 3:**
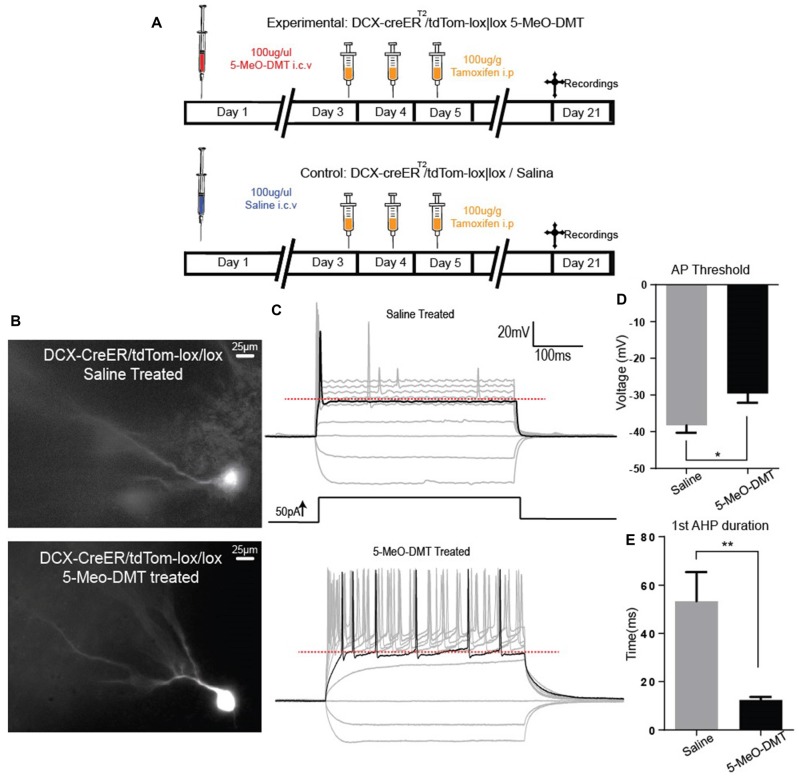
5-Meo-DMT injection alters afterhyperpolarization (AHP) duration and action potential (AP) threshold in immature hippocampus GC. **(A)** Animals received a dose of 100 μg of 5-MeO-DMT, followed by 100 μg/g of tamoxifen i.p. diluted in sesame oil 3 days after, daily for 3 days to allow cre recombination. Experiments were performed on day 21. **(B)** Photomicrography of a recorded tdTomato+ cells from control and 5-MeO-DMT-treated mouse. **(C)** Membrane potential changes in response to current steps, the black line denotes the trace in which the first AP was elicited, red dotted line denote AP threshold for that step. **(D)** Mean AP threshold. **(E)** Mean AHP duration. *p = 0.0216, **p = 0.0062.

**Figure 4:**
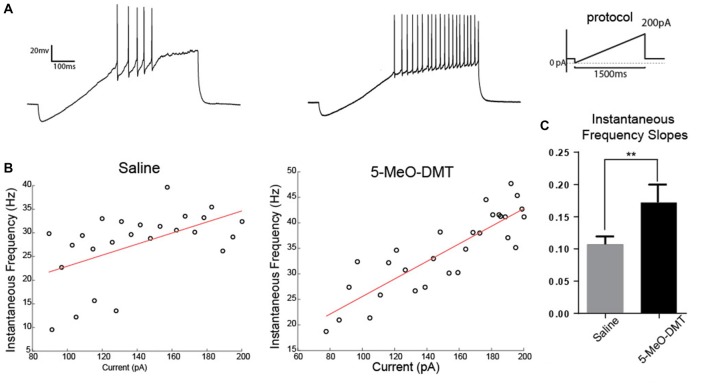
Young GC in 5-MeO-DMT-treated mice show a greater capacity for high frequency firing. **(A)** Membrane potential recording in response to a current ramp. **(B)** Linear regressions (ramp current vs. instantaneous AP frequency). **(C)** Average slopes (ramp current vs. instantaneous AP frequency relationship). **p = 0.0036.

Next, we recorded if 5-MeO-DMT can modify electrophysiological properties of immature DG GC, we performed whole cell patch clamp onto DCX-CreER^T2^::tdTom^lox/lox^ GC. In these experiments, DCX-CreER^T2^::tdTom^lox/lox^ mice were perfused 21 days after ICV injections to study morphological differences in dendritic processes (Figure 3A). Passive membrane and AP properties in response to a 500 ms-long 100 pA current step are shown in Table 1. Example membrane potential responses for a tdTomato+ cell from saline- and 5-MeO-DMT-injected mouse is shown in Figures 3B,C. Cells from 5-MeO-DMT-treated animals exhibited higher AP threshold (Saline: −38.25 ± 2.03 mV *n* = 8 cells/3 animals; 5-MeO-DMT: −29.60 ± 2.51 mV, *n* = 11 cells/3 animals, *p* = 0.022, unpaired *t-*test, Figure 3D). These cells also displayed a shorter AHP potential duration (Saline: 53.33 ms ± 12.04 ms *n* = 9 cells/3 mice; 5-MeO-DMT: 12.40 ms ± 1.302 ms, *n* = 8 cells/3 mice, *p* = 0.006, *t*-test, Figure 3E) associated to a single fire pattern, five from eight saline treated cells displayed single fire pattern vs. none from 5-MeO-DMT treated group. AP threshold was defined as the voltage in which the rate of rise reaches a value superior to 20 mv/ms. We then applied current ramps (−50 pA to 200 pA in 1.5 s) in order to elucidate differences in fast activated currents between the two experimental groups. Example membrane potential responses to the current ramp is shown in Figure 4A. Newborn GC from 5-MeO-DMT-treated mice showed a greater linear dependency between injected current and AP instantaneous frequency (Figures 4A,B). The slope from the linear regression ramp current (in pA) vs. AP instantaneous was equal to 0.11 ± 0.01 Hz/pA for controls and 0.17 ± 0.03 Hz/pA for 5-MeO-DMT group (*n* = 8 cells/three animals and *n* = 6 cells/three animals, respectively, *p* = 0.03, *t*-test, Figure 4B). We have also patched randomly chosen tdTomato+ cells form DCX-CreER^T2^::tdTom^lox/lox^ treated with saline (20 cells/four animals) or 5-MeO-DMT (16 cells/four animals) to test whether there was any cell in each group that did not fire in response to current injection. Three cells in the saline group (3/20 cells) did not fire APs while all cells fire APs in animals pre-treated with 5-MeO-DMT (*p* < 0.0001, *z* test). This data suggests that young GC from 5-MeO-DMT-injected mice show a higher degree of maturation than cells from control animals.

**Table 1:** Passive and active membrane properties extracted from tdTomato+ (DCX-Cre::tdTom^lox/lox^) granule cells (GC) across treatments.

	**Saline Treated**	**5-Meo-DMT Treated**
**Passive membrane properties**		
Input resistence (MΩ)	389.6 ± 70.06 *N* = 9	476 ± 32.55 *N* = 11
Baseline (mV)	−67.02 ± 3.340 *N* = 9	−70.74 ± 2.969 *N* = 11
ISI (ms)*	0.0571 ± 0.01204 *N* = 4	0.04587 ± 0.005734 *N* = 6
Number of spikes*	13.70 ± 4.386 *N* = 4	18.97 ± 2.470 *N* = 6
**Action potential properties**		
(+) Peak Amplitude (mV)	91.11 ± 3.971 *N* = 9	87.15 ± 3.663 *N* = 11
Rise time (ms)**	12.90 ± 6.359 *N* = 9	19.26 ± 2.099 *N* = 10
Latency (ms)***	128.5 ± 15.93 *N* = 9	166.4 ± 22.49 *N* = 11
Rate of rise (mv/ms)^#^	86.92 ± 7.910 *N* = 9	80.99 ± 7.937 *N* = 11
AP Threshold (mv)^##^	2.711 ± 14.83 *N* = 9	−31.87 ± 3.010 *N* = 11
AP Half Width (ms)	0.5689 ± 0.07323 *N* = 9	0.5218 ± 0.04441 *N* = 11
AP AHP Amplitude (mv)	−10.73 ± 2.317 *N* = 8	−6.324 ± 0.8275 *N* = 11
AP AHP Duration (ms)	53.33 ± 12.04 *N* = 9	12.40 ± 1.302 *N* = 8

*Interval interspikes and no. of spikes calculated from current ramp protocol (example show in Figure 4) all other variables were extracted from the first action potential (AP) of the Current steps protocol (as depicted in Figure 3); **Rise time stated as the time taken for the signal to go from 5% to 90% of its peak; ***Latency defined as the time between the beginning of the current step protocol and the 5% of the first peak (all protocols have 100 ms delay); ^#^Rate of Rise, defined as the maximum rate of change during the rising phase of the signal; ^##^AP threshold defined as the voltage point where the rate of rise reach a value superior to 20 mV/ms. AP half Width defined as the time taken to the signal rise from AP threshold to 50% its peak; AP after hyperpolarization (AHP) duration defined as the time beginning from 90% of AP peak until the voltage reach the value of 0% the peak again.

**Figure 5:**
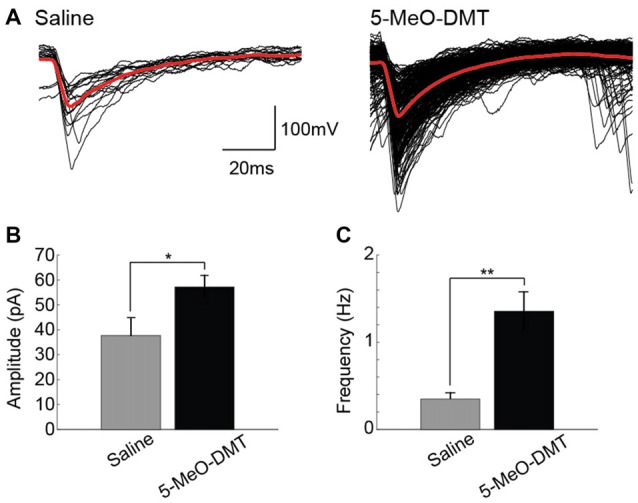
Young GC in 5-MeO-DMT-treated show a higher frequency of spontaneous excitatory postsynaptic potentials. **(A)** Examples of detectedspontaneous excitatory postsynaptic currents (sEPSCs; in 2 min recordings) cells from saline- and 5-MeO-DMT-treated mice. **(B)** Mean absolute sEPSC amplitude for saline- and 5-MeO-DMT-treated mice. *p = 0.03. **(C)** Average slopes (ramp current vs. instantaneous AP frequency relationship). **p = 0.001.

We have then recorded sEPSCs from six mice (three in each group, 18 cells) treated or not with 5-MeO-DMT (Figure 5A). While no difference in mean sEPSC half-width was found, sEPSC amplitude was greater in tdTomato+ cells of 5-MeO-DMT-treated mice im comparison to controls (57.15 ± 4.68 pA vs. 37.72 ± 6.60 pA, respectively, *p* = 0.03, *t*-test, Figure 5B). Also, the frequency of sEPSC was drastically increased in tdTomato+ GC of 5-MeO-DMT-treated mice when compared to controls (1.35 ± 0.22 Hz vs. 0.35 ± 0.07 Hz, respectively, *p* = 0.002, *t*-test, Figure 5C). These results indicate that newborn neurons from 5-MeO-DMT-treated mice are more prone to receive synaptic inputs from other DG cells.

**Figure 6:**
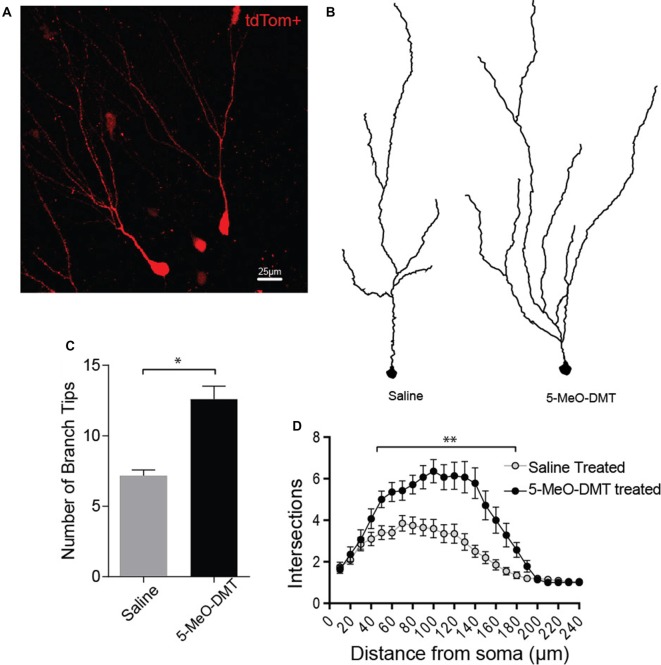
Single dose of 5-MeO-DMT increases dendritic complexity in young DG GC. **(A)** Sample image showing a tdTomato+ (CreER^T2^/tdTom^lox/lox^ mouse) granule cell with visible dendritic processes. **(B)** Vectorial reconstruction of tdTomato+ granule cell. **(C)** Mean number of branch tips of GC across treatments. **(D)** Sholl analysis comparing the dendritic complexity between two treatments with increasing radial distance from soma. *p = 0.0001, **p < 0.05.

We then tested if 5-MeO-DMT also alters morphological maturation of newborn GC (tdTomato+ neurons, Figure 6A). We first traced cells using an ImageJ plugin (see “Materials and Methods,” Section Figure 6B) to later perform morphological analysis of dendrites. Number of branches in dendrite tree between treatment groups were different (saline treated: 7.15 ± 0.4247 dendritic branches, *n* = 20 cells from six mice; 5-MeO-DMT treated: 12.6 ± 0.916 dendritic branches, *n* = 15 cells from six mice, *p* = 0.0001, unpaired *t*-test, Figure 6C). We then tested the same cells for dendritic complexity relative to cell nucleus, and the experimental group shows a higher number of intersections in the 50–170 μm range of distance from soma when compared to saline (saline intersect values: 50 μm, 3.4 ± 0.336; 60 μm, 3.4 ± 0.303; 70 μm, 3.850 ± 0.372; 80 μm, 3.750 ± 0.403; 90 μm, 3.650 ± 0.399; 100 μm, 3.6 ± 0.444; 110 μm, 3.350 ± 0.431; 120 μm, 3.350 ± 0.460; 130 μm, 2.950 ± 0.400; 140 μm, 2.5 ± 0.295; 150 μm, 2.2 ± 0.287; 160 μm, 1.850 ± 0.244; 170 μm, 1.55 ± 0.185, *n* = 20 cells from six mice; 5-MeO-DMT intersect values: 50 μm, 5 ± 0.406; 60 μm, 5.357 ± 0.464; 70 μm, 5.429 ± 0.456; 80 μm, 5.714 ± 0.450; 90 μm, 6.071 ± 0.559; 100 μm, 6.357 ± 0.561; 110 μm, 6.071 ± 0.606; 120 μm, 6.143 ± 0.653; 130 μm, 6.071 ± 0.752; 140 μm, 5.786 ± 0.735; 150 μm, 4.714 ± 0.699; 160 μm, 4.0 ± 0.629; 170 μm, 3.286 ± 0.569, *n* = 15 cells from six mice. The significance for each intersection comparison was respectively: 50 μm, *p* = 0.0291; 60 μm, *p* = 0.0028; 70 μm, *p* = 0.0309; 80 μm, *p* = 0.0028; 90 μm, *p* = 0.0001; 100 μm, *p* = 0.0001; 110 μm, *p* = 0.0001; 120 μm, *p* = 0.0001; 130 μm, *p* = 0.0001; 140 μm, *p* = 0.0001; 150 μm, *p* = 0.0001; 160 μm, *p* = 0.0007; 170 μm, *p* = 0.0128, Two-way ANOVA, Figure 6D). Taken together, these results suggest that 5-MeO-DMT accelerates dendritic growth toward a morphology of a fully mature DG granule neuron.

## Discussion

In this work we showed that a single dose of 5-MeO-DMT increases proliferation of neural progenitors and accelerates the maturation of newborn GC. We first used BrdU staining to show that 5-MeO-DMT treatment increases proliferation in the DG Next, we used an inducible Cre recombinase line under the control of a marker of neurogenesis (DCX) crossed with a fluorescent reporter to identify newborn neurons. In Figure 2 we show that the total number of DCX::tdTom+ cells in the ventral hippocampus of adult mice are increased, and this cells are likely DG GC, as was *post hoc* confirmed by electrophysiological, and morphological data that those cells are indeed neurons. Dendritic trees of newborn neurons from 5-MeO-DMT-treated mice were significantly more complex (with more branches and a higher number of intersections) when compared to saline-treated mice. AP threshold was lower and AHP potential was longer in newborn cells from 5-MeO-DMT-treated mice compared to controls.

The higher number of BrDU+ cells indicate that a larger number of cells are entering in the S-phase of cell-cycle (Taupin, 2007), but cannot elucidate the type of progenitor cell that is being affected. Studies using antibodies against GFAP, nestin and Sox2, might confirm if those BrdU+ cells are indeed RGL cells, the neural stem progenitors cells from adult DG (Kempermann et al., 2004; Lagace et al., 2007; Rizzino, 2009). Also, future experiments may confirm whether the increase in BrdU+ cells following 5-MeO-DMT injection is due to the lengthening of S-phase or a higher recruitment of RGL (for example, using antibodies against ki67 or MCM2 associated with BrDU; Kee et al., 2002).

The choice of a single dose treatment, was made to address the gap between the molecular mechanisms, subjective and hormonal effects underlying *Ayahuasca* acute administration to depression diagnosed patients (dos Santos et al., 2016; Sanches et al., 2016; Galvão et al., 2018; Palhano-Fontes et al., 2018). The bulk of *Ayahuasca* tea, are composed of several psychoactive substances including DMT analogs and MAOi (Frecska et al., 2016; Morales-García et al., 2017). The scope of present study is to unveil the effect of the 5-MeO-DMT, without adding any bias, due to other psychoactive compounds. To study the specific contribution of the 5-MeO-DMT to the adult neurogenic process, we needed to isolate the effect of the 5-MeO-DMT from other psychoactive components. In *Ayahuasca* tea the DMT is administrated with MAOi, in order to avoid tryptamines degradation. Using oral or intraperitoneal administration without MAOi may reduce the availability of 5- MeO-DMT to the central nervous system, since the monoamine oxidase will readily destroy any tryptamine, in the bloodstream, guts and also in the brain (Halberstadt et al., 2008; Halberstadt, 2016; Morales-García et al., 2017). Since 5-MeO-DMT can easily be degraded, we chose to deliver the 5-MeO-DMT i.c.v. to reduce the chemical inactivation prior to the arrival of the molecule to the brain. Additionally, it has been reported elsewhere that the harmine *per se* can increase neurogenesis, at least *in vitro* cultured hippocampal cells (Morales-García et al., 2017).

Increased proliferation after 5-MeO-DMT injection does not indicate neuronal commitment (Canales, 2016). Thus, we performed histological analysis in DCX-CreER^T2^::tdTom^lox/lox^ mice injected with 5-MeO-DMT. Our results indicate a greater number of DCX::tdTom+ cells in the ventral hippocampus of 5-MeO-DMT treated animals, showing that the total numbers of neuron that reach neuronal maturity are also increased, in addition to the initial increase in proliferation right after 5-MeO-DMT injection as evinced by our proliferation assay Figure 1. Serotonin has been shown to increase granule cell proliferation in the adult DG (Brezun and Daszuta, 2000). However, serotonin does not seem to affect specialization of newborn cells in the SGZ (Brezun and Daszuta, 2000). Our results, on the other hand, suggest that 5-MeO-DMT not only has a positive effect on proliferation and survivability, but also on the maturation of GC. Hence, our results imply that the positive effect of 5-MeO-DMT in adult neurogenesis differs from that of serotonin alone.

Our current-clamp recordings indicate that young neurons from 5-MeO-DMT-treated mice show faster maturation than cells from control animals. Mature GC show a higher AP threshold and are able to fire in higher frequencies (Schmidt-Hieber et al., 2004). These differences in maturation were also found in the morphology of dendritic trees. Dendritic complexity is a major indicative of cell maturation (Schmidt-Hieber et al., 2004; Ohira and Miyakawa, 2011). Cells from animals submitted to a single 5-MeO-DMT injection showed dendrites with more branches and intersections. Interestingly, chronic antidepressant therapy also accelerates the maturation of dendrites (Ohira and Miyakawa, 2011). Future studies should address how tryptamine analogs affect the temporal expression of voltage-dependent currents. Our preliminary results indicate, for example, that the hyperpolarizing-activated current (Leão et al., 2011), *I*_h_, is larger in novel GC in animals injected with 5-MeO-DMT when compared with saline Also, it will be interesting to examine changes in Cl^−^ reversal potential as GC show a depolarized potential until adolescence (Chiang et al., 2012).

Dorsal Raphe Nucleus profusely targets the SGZ (Kosofsky and Molliver, 1987) but a previous work have shown that lowering serotonin levels in the brain can increase neurogenesis (Song et al., 2016). Yet, serotonin agonists and serotonin uptake inhibitors seem to increase neurogenesis (Ohira and Miyakawa, 2011; Surget et al., 2011). Hence, specific 5HT receptors might be involved in neurogenesis modulation. 5-HT_1A_, 5-HT_2A_ and 5-HT_2C_, 5-MeO-DMT targets, are all expressed in the DG (Allen Institute for Brain Science^1^, experiments n°: 79394355, 81671344 and 71393424, respectively). While 5-MeO-DMT is a strong 5-HT_2A_ and 5-HT_2C_ agonist, this compound acts in other receptors (with much lower potency). Hence, we cannot affirm that the effect of 5-MeO-DMT in neurogenesis occurs through 5-HT_2A_ and 5-HT_2C_ receptors. Future studies using agonists and antagonists are necessary for dissecting the molecular mechanism of 5-MeO-DMT action in neurogenesis.

In conclusion, we show here that a single dose of 5-MeO-DMT can increase proliferation, survivability and accelerate maturation of newborn neurons in the DG. To our knowledge, this work was the first to demonstrate a direct effect of a naturally occurring psychoactive compound in adult neurogenesis. New lines of investigation have suggested that serotoninergic hallucinogens can significantly improve severe depression and anxiety (Reiche et al., 2018). Thus, the effect of 5-MeO-DMT in modulating neurogenesis could throw light on the mechanism behind the beneficial effects of hallucinogenic compounds in mood disorders.

## Author Contributions

RVLC did experiments, analyzed data and wrote the article. TM analyzed data. RL analyzed data and wrote the article.

RVLC and RL did experiments, analyzed data and wrote the article. TM analyzed data. LLP did experiments.

## Conflict of Interest Statement

The authors declare that the research was conducted in the absence of any commercial or financial relationships that could be construed as a potential conflict of interest.

## Footnotes

^1^http://www.alleninstitute.org/
