# Corrigendum: A Single Dose of 5-MeO-DMT Stimulates Cell Proliferation, Neuronal Survivability, Morphological and Functional Changes in Adult Mice Ventral Dentate Gyrus

**DOI:** 10.3389/fnmol.2019.00079

**Published:** 2019-04-04

**Authors:** Rafael Vitor Lima da Cruz, Thiago C. Moulin, Lyvia Lintzmaier Petiz, Richardson N. Leão

**Affiliations:** ^1^Neurodynamics Lab, Brain Institute, Federal University of the Rio Grande do Norte, Natal, Brazil; ^2^Institute of Medical Biochemistry, Federal University of Rio de Janeiro, Rio de Janeiro, Brazil; ^3^Developmental Genetics, Department of Neuroscience, Uppsala University, Uppsala, Sweden

**Keywords:** 5-MeO-DMT, adult neurogenesis, patch clamp, psychedelics, dentate gyrus granule cells

In the original article, there was an error. We mistakenly stated that 5-MeO-DMT is part of the ayashuasca brew.

A correction has been made to the ***Introduction***, paragraph one:

“Psychoactive tryptamines are a class of molecules that act as a neurotransmitter in the vertebrate brain (Jacob and Presti, 2005). N,N-dimethyltryptamine, (DMT) and analogues, are closely related to 5-methoxy- N,N-dimethyltryptamine (5-MeO-DMT), they can be found in a great variety of plants in South America, with an even greater diversity of chemical analogs (Geyer et al., 2010; Greene, 2013). 5-MeO-DMT is a serotonin agonist that acts in a non-selective manner in 5-HT_2A_ >5-HT_2C_ >5-HT_1A_ receptors (Szabo et al., 2014). However, the N-N-DMT has been reported elsewhere to also acts in many glutamate, dopamine, and acethylcholine receptors (Carbonaro and Gatch, 2016). It would be interesting to know whether the 5-MeO-DMT have the same effect as its analogue on those receptors. The 5-MeO-DMT is analogous of the N,N-DMT, one of the main active ingredients of *Ayahuasca*, a millenarian decoction used as a sacrament by south American indigenous tribes, known to induce powerful hallucinogenic states when administered with monoamine oxidase inhibitors (MAOI; Araújo et al., 2015). At present, *Ayahuasca* is used by many syncretic churches ritualistically, as a way to heal many physical and mental illnesses with or without scientific knowledge about the effects (Frecska et al., 2016). Recent studies also suggest that *Ayahuasca* can potentially treat recurrent depression (Osório Fde et al., 2015; Sanches et al., 2016) even in a placebo controlled frame (Palhano-Fontes et al., 2018).”

Additionally, a correction has been made to the ***Discussion***, paragraph three:

“The choice of a single dose treatment, was made to address the gap between the molecular mechanisms, subjective and hormonal effects underlying *Ayahuasca* acute administration to depression diagnosed patients (dos Santos et al., 2016; Sanches et al., 2016; Galvão et al., 2018; Palhano-Fontes et al., 2018). The bulk of *Ayahuasca* tea, are composed of several psychoactive substances including DMT analogs and MAOi (Frecska et al., 2016; Morales-García et al., 2017). The scope of present study is to unveil the effect of the 5-MeO-DMT, without adding any bias, due to other psychoactive compounds. To study the specific contribution of the 5-MeO-DMT to the adult neurogenic process, we needed to isolate the effect of the 5-MeO-DMT from other psychoactive components. In *Ayahuasca* tea the DMT is administrated with MAOi, in order to avoid tryptamines degradation. Using oral or intraperitoneal administration without MAOi may reduce the availability of 5-MeO-DMT to the central nervous system, since the monoamine oxidase will readily destroy any tryptamine, in the bloodstream, guts and also in the brain (Halberstadt et al., 2008; Halberstadt, 2016; Morales-García et al., 2017). Since 5-MeO-DMT can easily be degraded, we chose to deliver the 5-MeO-DMT i.c.v. to reduce the chemical inactivation prior to the arrival of the molecule to the brain. Additionally, it has been reported elsewhere that the harmine *per se* can increase neurogenesis, at least *in vitro* cultured hippocampal cells (Morales-García et al., 2017).”

The authors apologize for this error and state that this does not change the scientific conclusions of the article in any way. The original article has been updated.
